# Relationship between Alcohol Hangover and Physical Endurance Performance: Walking the Samaria Gorge

**DOI:** 10.3390/jcm9010114

**Published:** 2019-12-31

**Authors:** Joris C Verster, Aikaterini Anogeianaki, Darren Kruisselbrink, Chris Alford, Ann-Kathrin Stock

**Affiliations:** 1 Division of Pharmacology, Utrecht Institute for Pharmaceutical Sciences (UIPS), Utrecht University, 3584CG Utrecht, The Netherlands; j.c.verster@uu.nl (J.C.V.); kanogeianaki@gmail.com (A.A.); 2 Institute for Risk Assessment Sciences (IRAS), Utrecht University, 3584CM Utrecht, The Netherlands; 3 Centre for Human Psychopharmacology, Swinburne University, Melbourne, VIC 3122, Australia; 4 Centre of Lifestyle Studies, Acadia University, Wolfville, NS B4P 2R6, Canada; darren.kruisselbrink@acadiau.ca; 5 Psychological Sciences Research Group, University of the West of England, Bristol BS16 1QY, UK; chris.alford@uwe.ac.uk; 6 Cognitive Neurophysiology Department of Child and Adolescent Psychiatry, Faculty of Medicine of the TU Dresden, University of Dresden, D-01307 Dresden, Germany

**Keywords:** physical performance, hiking, walking, water consumption, alcohol, hangover, sleep, Samaria Gorge

## Abstract

Alcohol hangover is a potentially debilitating state. Several studies have demonstrated that it does not seem to impair strength or short-term endurance, but its effects on continuous exercise performance/long-term endurance have never been investigated. Therefore, the aim of the current study was to assess hiking performance of participants who walked the 15.8 km Samaria Gorge in Crete, Greece. Participants completed a survey in the morning before walking the Gorge, and in the afternoon after completion of the walk. Demographics, data on previous evening alcohol consumption, sleep, hangover symptoms, and walking performance were assessed. Data from *N* = 299 participants with a mean (SD) age of 38.9 (11.0) years were analyzed. *N* = 223 participants (74.6%) consumed alcohol the evening before walking the Samaria Gorge, and *N* = 176 (78.9%) of those reported a hangover. They consumed a mean (SD) of 3.0 (1.8) alcoholic drinks (10 g alcohol each) with a corresponding next-morning hangover severity of 4.6 (2.4) on a 0–10 scale. Participants with a hangover reported feeling significantly more exhausted after the walk compared to participants with no hangover. The groups did not significantly differ in duration of the walk, and the number and duration of breaks. Overall hangover severity, assessed either before, during, or after walking the Samaria Gorge was not significantly correlated with any walking outcome. In conclusion, hungover participants experienced significantly more exhaustion when performing physical activity at the same level as non-hungover participants.

## 1. Introduction

The alcohol hangover is defined as the combination of mental and physical symptoms experienced the day after a single episode of heavy drinking, starting when the blood alcohol concentration (BAC) approaches zero [1]. A growing body of evidence shows that cognitive functioning and mood are negatively affected in the hangover state [2,3], which may result in impaired daily activities such as job performance [4,5], riding a bicycle [6], or driving a car [7,8,9]. 

Much less research has been devoted to the possible impact of the alcohol hangover on physical performance. Van Schrojenstein Lantman et al. [10] investigated the impact of individual hangover symptoms on physical performance. Participants reported that being tired, sleepiness, headache, nausea, and weakness had the greatest impact on their physical performance in the hangover state. However, the few studies that have investigated physical performance in the hangover state to date have failed to find significant effects. In 30 male students, Nelson et al. [11] examined various physical performance activities, including grip strength, a 45-s bicycle ergometer test assessing the maximum number of revolutions against 10 Lb resistance, softball throwing, a vertical jump, and push-ups. Performance in the hangover state did not differ significantly from performance on the control day for any of the tests. Karvinen et al. [12] examined physical performance in 30 firemen and policemen on grip strength, back lift, vertical jump test and a 5-min bicycle ergometer test on a hangover and control day. In this study, also, no significant performance differences were found between the hangover and control day. In a more recent study, Kruisselbrink et al. [13] examined physical activity in the hangover state in 12 females. They performed a 6-min submaximal treadmill run at 6 miles per hour, followed by a run to exhaustion once the treadmill had been raised to a 7% grade. In addition, a grip strength test was conducted. Again, no significant performance difference was observed during the hangover state vs. control. 

Devenney et al. [14] assessed physical activity levels during the hangover state. Compared to an alcohol-free control day, activity levels were significantly reduced in the hangover state. In particular, the percentage of time spent on vigorous activity was significantly reduced and a significantly higher percentage of time was spent in a sedentary manner on the hangover day. No significant differences between the hangover and control day were found for time spent on light and moderate activity. Unfortunately, the type of activities participants engaged in was not recorded in this study. Nevertheless, the data show that the activity level is reduced to less demanding levels during the hangover state. Based on this observation, it is tempting to hypothesize that participants may be less capable of performing physical activity at vigorous levels during the hangover state, but more research is needed to verify this. 

It should be noted that physical performance in these studies was assessed with tests of relatively short duration and can often be categorized as assessing strength (e.g., grip strength) or balance (e.g., standing on one foot) or short-term endurance (e.g., a bicycle test). As of now, there are however no studies that have examined long-term endurance, i.e., physical performance effects over a longer time, such as a 10 km walk, long-distance running, playing a football match, or skiing. Furthermore, the sample sizes of these studies (12–30 subjects) were relatively small, which may have contributed to observing nonsignificant findings. Therefore, the aim of the current study was to investigate the effects of alcohol hangover on endurance performance within a naturalistic setting. To this extent, walking performance was examined in participants walking the 15.8 km Samaria Gorge, in Crete, Greece.

## 2. Materials and Methods

The study was conducted in the summer of 2019, at Crete, Greece. Participants who booked a tour to walk the Samaria Gorge were invited to participate in the study. They were recruited on the bus drive towards the entrance of the Samaria Gorge. Participants met the inclusion criteria if they were 18–65 years old and could understand and write English. A researcher was present to help clarify any questions that arose while completing the surveys. The study was conducted by researchers from Utrecht University. The survey was anonymous and participants did not receive an incentive for completing the surveys. The Ethics Committee of the Faculty of Social and Behavioral Sciences of Utrecht University granted ethical approval (approval code FETC17-061). *N* = 307 participants provided informed consent to take part in the study. 

### 2.1. The Walk

Data were collected on 9 different day-excursions to the Samaria Gorge between 23 July and 11 August 2019. Weather data for these days at the Samaria Gorge were obtained from www.worldweatheronline.com. On all days except one (partly cloudy), the weather was described as ‘sunny’. No rainfall was recorded on any of the excursions, the average humidity was 56% with a pressure of 1009 mb. The average temperature rose from 27.7 °C at 21:00 to 29.2 °C at 12:00, 28.9 °C at 15:00 and 27.2 °C at 18:00. A paired samples t-test revealed no significant temperature difference between individual test days and the mean 9-day temperature (*p* = 0.988).

**Figure 1:**
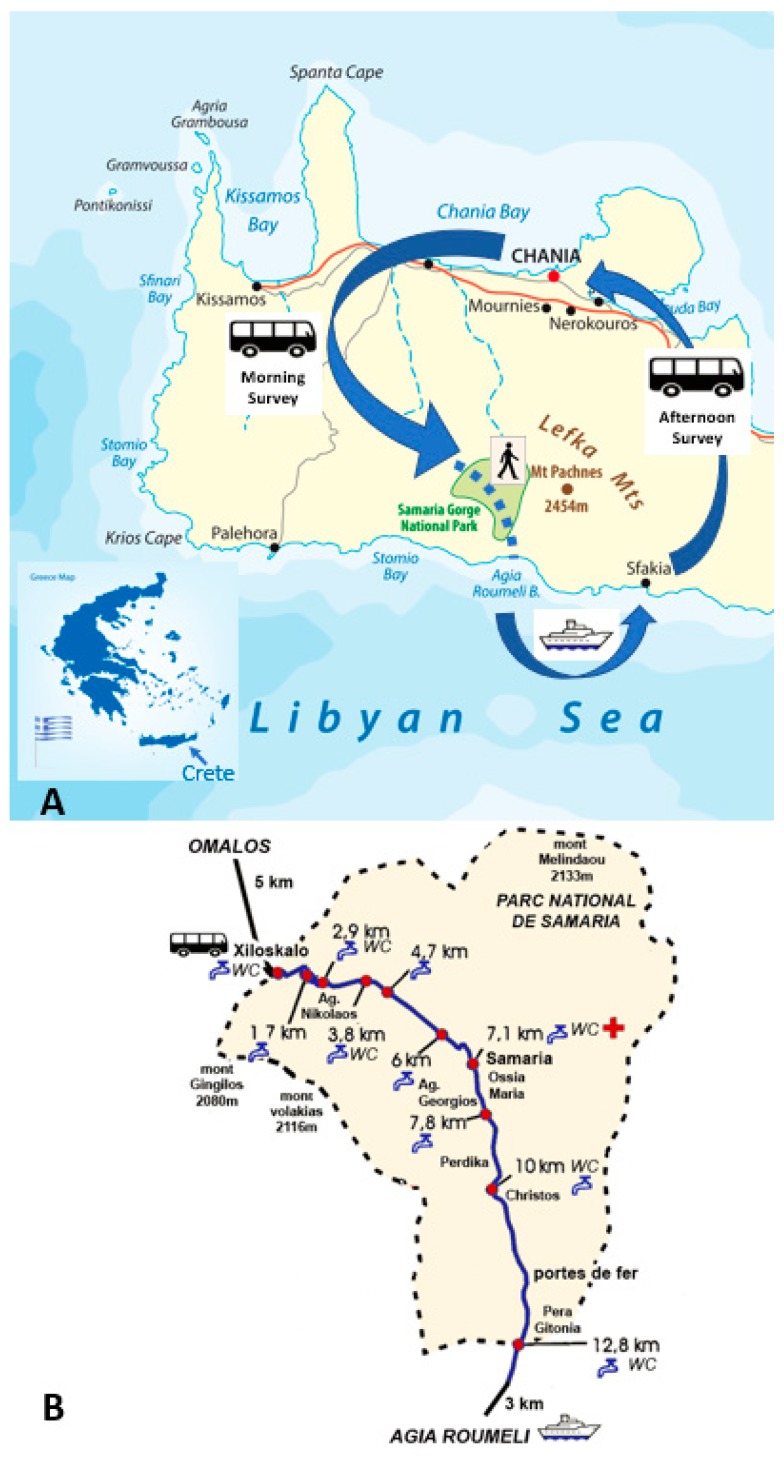
Schematic representation of the day tour and Samaria Gorge walk. (**A**) is adapted and used with permission from www.depositphotos.com. (**B**) is adapted and used with permission from Psarakis Travel Agency (www.psarakistravel.gr).

Figure 1A schematizes the day tour of the participants. They were picked up at their accommodation between 06:15 and 06:45 in the morning. Participants had booked their day-excursion to the Samaria Gorge either from Chania (*n* = 2 excursions), Rethymnon (*n* = 4 excursions) or Herakleion (*n* = 3 excursions). Depending on their city of residence, they arrived at the Samaria Gorge after a bus drive of approximately 2 to 3 h: Chania (08:35), Rethymnon (09:00) or Herakleion (09:15). From Hora Sfakia, all busses returned to the participants’ accommodations in the afternoon at 18:00. Figure 1B shows the several resting locations where participants could drink water during the walk. These are simple tap points, as there are no inhabited villages or stores from which to buy refreshments in the Samaria Gorge.

**Figure 2:**
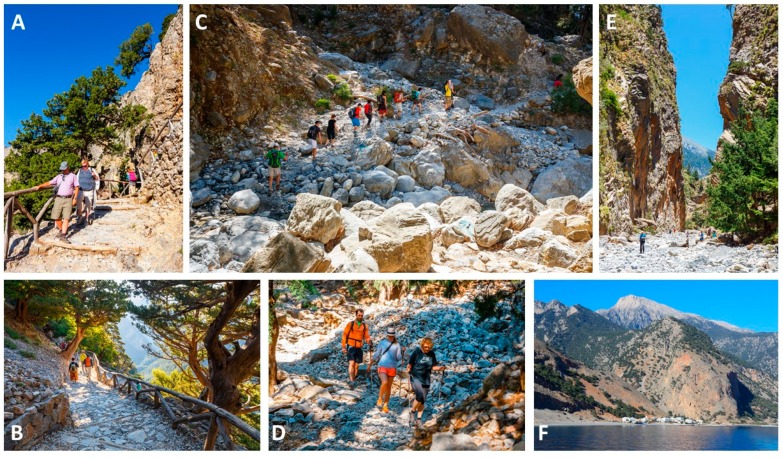
Impressions of the Samaria Gorge walk. (**A**,**B**) Over the first kilometers, participants descend a narrow switchback path, which is partly bordered by a wooden handrail. A large part of the walk is trough rough terrain, i.e., the slippery stones of a dried-up riverbed. In winter, the riverbed is filled with water, but in summer the river has mostly dried up. Participants cross the riverbed several times, sometimes aided by wooden improvised bridges. (**C**,**D**) there is no paved road but participants have to follow a trail through a rocky valley. Samaria is the main resting place for most participants, as this uninhabited tiny village contains benches in the shade, a water tap point and a medical post. Through the last part of the walk, the cliffs become higher and narrower. (**E**) At its narrowest point, called the “Gates”, the Samaria Gorge is approximately 4 m wide, while the cliffs rise over 300 m high. (**F**) Thereafter, the final fairly flat part of the walk ends in Agia Roumeli at the seaside. Participants can relax, eat and drink in the taverns, and recover from the walk. From Agia Roumeli, a ferry brings the participants to Hora Sfakia in about 1 h. From there, coaches leave at 18:00 to bring the participants back to their accommodation. Figures adapted and used with permission from www.depositphotos.com.

The Samaria Gorge walk is 15.8 km long. It starts in Xiloskalo at an altitude of 1230 m and ends in Agia Roumeli, a small village at the Libyan Sea. Agia Roumeli can only be reached by walking the Samaria Gorge or by boat. The actual walk through the Samaria Gorge is about 12.8 km long, and after the end of the gorge, it takes another 3 km of walking to reach Agia Roumeli. Hence, participants walk 15.8 km in total, starting at an altitude of 1230 m, slowly descending to sea level in Agia Roumeli. The first part of the walk is 3.8 km long with a 580 m descent, followed by 3.7 km with a 310 m descent. Thereafter, the decent is less steep (3.6 km with a decent of 170 m). The last 4.7 km has a decent of 170 m towards sea level. An impression of the Samaria Gorge walk is given in Figure 2. 

### 2.2. Data Collection

Two surveys were conducted. The first survey was completed in the bus, before walking the Samaria Gorge. Demographic information was collected. Participants provided information on the past evening’s alcohol consumption. They reported the number of alcoholic drinks consumed (European sizes were provided as examples, which contain 10 g alcohol each) as well as the start and stop time of drinking. Together with information on sex and weight, this allowed for the calculation of their estimated blood alcohol concentration (BAC) using a modified Widmark formula [15]. Being drunk/intoxicated was rated on an 11-point scale ranging from 0 (absent) to 10 (extreme) [16,17]. The number of smoked cigarettes was recorded as well, as previous research has shown an interaction between smoking and hangover [18]. To capture possible effects of sleep [14,19,20], total sleep time was recorded as well as the (consciously perceived) number of nightly awakenings. Sleep quality was rated on an 11-point scale ranging from 0 (very poor) to 10 (excellent) [21,22]. 

Physical activity levels were assessed with the IPAQ (International Physical Activity Questionnaire)—short form [23]. This measure assesses the intensity and duration of physical activity and sitting time that people do as part of their daily lives. The outcomes are presented in Metabolic Equivalent Task (MET)-minutes. MET minutes represent the amount of energy spent when engaged in a physical activity. One MET is what you expend when you are at rest. When calculating the MET-minutes per week for the different activity levels, the duration of activity is multiplied with 3.3 (walking), 4 (moderate physical activity) or 8 (vigorous physical activity). The assessments were made for usual physical activity at home, and for physical activity during their holiday in Crete.

Overall hangover severity before starting the walk was rated on a single item 11-point scale ranging from 0 (absent) to 10 (extreme) [24]. Using the same 11-point scale, the severity of a number of individual symptoms that are often related to hangover was also assessed. These included fatigue (being tired), sleepiness, thirst, headache, nausea, loss of appetite, dizziness, stomach pain, heart racing, weakness, anxiety, depression, tension/stress, and anger/hostility. The past year’s immune fitness was assessed with the Immune Status Questionnaire (ISQ) [25]. The ISQ consists of 7 items, including ‘common cold’, ‘diarrhea’, ‘sudden high fever’, ‘headache’, ‘muscle and joint pain’, ‘skin problems (e.g., acne and eczema)’ and ‘coughing’. The items are scored on a 5-point Likert scale stating how often the participants experienced these complaints during the past year, including ‘never’, ‘sometimes’, ‘regularly’, ‘often’, and ‘(almost) always’. The overall ISQ score ranges from 0 (poor) to 10 (excellent), with higher scores indicating a better immune fitness. Current immune fitness (in Crete) was assessed using the 1-item perceived immune functioning scale [26,27]. In a similar way, current ‘physical fitness’ and ‘mental fitness’ were assessed. The scores on these scales ranged from 0 (very bad) to 10 (very good). Finally, participants estimated the amount of effort it would take them to walk the Samaria Gorge on an 11-point scale ranging from 0 (absolutely no effort) to 10 (extreme effort). 

The surveys were collected by the researcher once completed. When the participants arrived at the entrance of the Samaria Gorge and started their walk, the bus returned by road and drove towards Hora Sfakia, a small seaside city where the participants were picked up at 18:00 to return by bus to their accommodation. The second survey was completed at the end of the day (between 18:00 and 19:00), during the bus drive to their accommodation. In the second survey, participants reported how they experienced walking the Samaria Gorge. Data were collected on the total duration of the walk, the number and duration of breaks, and the amount of water consumed while walking. Participants rated the amount of effort it took them to walk the Samaria Gorge on an 11-point scale ranging from 0 (absolutely no effort) to 10 (extreme effort). Their level of exhaustion was assessed on an 11-point scale ranging from 0 (absolutely not) to 10 (extremely exhausted). Using the scale described above, overall hangover severity was assessed for two additional time frames: (1) retrospectively for how they were during the walk, and (2) in real-time after the walk (when travelling on the bus to their accommodation). Of note, anxiety, depression, tension/stress, and anger/hostility were omitted from the ‘during the walk’ assessment. Finally, the number of alcoholic drinks consumed and cigarettes smoked after walking the Samaria Gorge were recorded, and subjective intoxication was rated as described above [16,17]. 

### 2.3. Statistical Analysis

All surveys were anonymous. However, because date of birth and sex were entered on both the morning and afternoon survey, they could be matched with each other. Data from participants who used drugs, or who did not the complete the questions on previous evening alcohol consumption were omitted from the statistical analysis. Statistical analyses were conducted with SPSS (IBM Corp. Released 2013. IBM SPSS Statistics for Windows, Version 25.0. Armonk, NY, USA: IBM Corp.). Mean and standard deviation (SD) were computed for each variable. Most data in the study did not follow a normal distribution. Therefore, nonparametric statistics were used to analyze the data. As a nonparametric analog of analysis of variance (ANOVA), the Kruskal–Wallis test was used to compare outcomes of the ‘no alcohol’, ‘no hangover’, and hangover’ groups. If significant, Independent Samples Mann–Whitney U tests (the nonparametric analog of independent t-tests) were used to make paired comparisons between two individual groups (e.g., compare the ‘hangover’ versus ‘no hangover’ group). To compare assessments made within subjects (e.g., before versus after walking), Related Samples Wilcoxon Signed Rank tests were conducted (the nonparametric analog of paired t-tests). Spearman’s rho correlations were used to compute correlations. Results were considered statistically significant if *p* < 0.05. In case of multiple related comparison (i.e., hangover symptoms), a Bonferroni’s correction was applied to account for multiplicity. Linear stepwise regression analyses (for which independent variables do not need to be normally distributed or continuous) were conducted to determine which of the individual variables assessed in this study were significant predictors of walking outcomes.

## 3. Results

### 3.1. Demographics and Physical Activity Levels

*N* = 307 participants provided consent to participate in the study. One subject used illicit drugs and her data was excluded from the analysis. *N* = 7 other participants did not report on previous evening alcohol consumption, and these incomplete datasets and were also excluded from the analyses. Data for the remaining *N* = 299 participants were included in the analyses. Except for N=6 participants from Canada (2%), all participants originated from 17 European countries. Most participants came from Germany (14.0%), the UK (12.5%), The Netherlands (11.4%), Poland (7.0%), Belgium (6.7%), and Greece (6.1%). *N* = 223 participants (74.6%) consumed alcohol the evening before walking the Samaria Gorge, and *N* = 176 (78.9%) of those reported a hangover score greater than zero in the morning. Their demographics are summarized in Table 1.

Table 1 shows that at home, participants in the ‘hangover’ group consumed significantly more alcohol (*p* < 0.0001) compared to the ‘no hangover’ group and ‘no alcohol’ group. The evening before the walk, participants with a hangover smoked significantly more cigarettes (*p* < 0.0001), and reported a significantly reduced sleep quality (*p* < 0.0001) and sleep duration (*p* = 0.022) than the ‘no hangover’ group. Although some differences between the intensity levels were observed, total physical activity at home and in Greece did not significantly differ between the groups.

### 3.2. Drinking Characteristics and Hangover Symptom Severity

Alcohol consumption characteristics the evening before their Samaria Gorge excursion for drinkers with and without a hangover on the day of their walk are summarized in Table 2.

Table 2 shows that, the evening before their excursion, participants in the ‘hangover’ group consumed on average 3.0 alcoholic drinks (European size, 10 g alcohol each). Although this seems a low amount of alcohol in comparison to, for example, student samples [24], it equates to half their usual weekly alcohol consumption (see Table 1). Overall hangover severity declined during the day. Participants were allocated to the ‘no hangover’ group when overall hangover severity was rated zero in the morning. However, some of these participants did report positive hangover scores during or after the walk. Hence, the mean hangover severity is not zero in the ‘no hangover’ group for these assessments. Severity scores for individual hangover symptoms, rated before, during (retrospectively), and after walking the Samaria Gorge are summarized in Table 3. 

In the morning, hangover symptom severity scores were usually significantly higher in the ‘hangover’ group compared to the ‘no hangover’ group. During and after the walk, no significant differences were found between the ‘hangover’ group and ‘no hangover’ group.

The hangover group experienced a significant reduction in severity scores during the walk for sleepiness, headache, nausea, dizziness, and stomach pain. No significant differences were observed between individual symptom severity assessment ‘during’ or ‘after’ the walk, indicating that the experience of symptom severity had plateaued during the walk. 

Although often not considered in hangover research, various symptoms experienced during the hangover state are also experienced by participants who reported having no hangover or consumed no alcohol at all, thus reporting overall hangover severity scores of zero. Although their severity scores are usually low and fairly constant, ratings for fatigue, thirst, and weakness significantly increased during the walk in both the ‘no alcohol’ and ‘no hangover’ group (*p* < 0.0001). Compared to the morning assessments, there was also a significant increase in heart racing severity during the walk in the ‘no hangover’ group. Again, no significant differences between ‘during’ and ‘after’ walking assessments were observed in these groups.

Table 3 further summarizes alcohol consumption and smoking data that occurred after the completion of walking the Samaria Gorge. Across all groups, alcohol consumption, smoking, and subjective intoxication levels were relatively low.

As this alcohol consumption and smoking occurred after the walk while waiting for the bus to return to their apartments/hotel, they have no impact on the dependent variables in this study (i.e., the walking outcomes). Their effects on hangover severity assessed after the walk are described in Section 3.7.

### 3.3. Walking Performance

Table 4 summarizes the walking outcomes and fitness measures of the three groups.

Participants in the ‘hangover’ group felt significantly more exhausted after walking the Samaria Gorge compared to participants in the ‘no hangover’ group (*p* = 0.004). The latter was anticipated by participants of the ‘hangover’ group, as they rated the expected effort to complete the walk as significantly higher than the ‘no hangover’ group (*p* < 0.0001). However, actual effort scores did not significantly differ between the ‘hangover’ group and ‘no hangover’ group (*p* = 0.136). Also, participants with a hangover consumed significantly more water compared to participants with no hangover (300 mL more on average, *p* = 0.043) and those who consumed no alcohol (400 mL more on average).

Interestingly, the immune fitness rating before walking was significantly better in the ‘no hangover’ group (*p* < 0.0001) compared to the ‘hangover’ group and the ‘no alcohol’ group (*p* < 0.0001). In the ‘hangover’ group, the immune fitness rating did not significantly correlate with overall hangover severity (*r* = 0.101, *p* = 0.184). 

### 3.4. Correlates of Walking Performance

Overall hangover severity, assessed either before, during (in retrospect), or after walking the Samaria Gorge was not significantly correlated with any walking outcome. 

Linear stepwise regression models were computed, including sex, age, BMI, weekly alcohol consumption, Total MET/week home and Crete, time spent sitting home and Crete, ISQ, general health rating, immune/menta/physical fitness in morning, number of cigarettes smoked, overall hangover severity (but NOT all individual hangover symptoms) in the morning, total sleep time, nightly awakenings, sleep quality, all drinking outcomes, and group (‘no alcohol’, ‘no hangover’, ‘hangover’). The models are summarized in Table 5.

### 3.5. Sex Differences

Of the participants with a hangover, *N* = 105 were men and *N* = 71 were women. Only a few sex differences were observed. With regards to demographics, women had a significantly lower BMI than men (22.8 ± 1.9 vs. 24.9 ± 2.3 kg/m^2^, *p* < 0.0001), spent less time on walking at home (2918 ± 1585 vs. 3566 ± 1911 MET-minutes, *p* = 0.035), reported less physical activity (total METs per week) at home (5014 ± 2974 vs. 6390 ± 3981 MET-minutes, *p* = 0.037), and reported a significantly lower weekly alcohol consumption at home (3.9 ± 1.8 vs. 7.4 ± 3.4 alcoholic drinks, *p* < 0.0001). Women’s reported past year immune fitness was significantly higher than men’s (9.1 ± 1.4 vs. 8.5 ± 1.6, *p* = 0.004). 

The evening before the walk, women smoked significantly fewer cigarettes (4.3 ± 5.8 vs. 6.6 ± 6.9 cigarettes, *p* = 0.026), and consumed significantly less alcohol (2.3 ± 1.3 vs. 3.5 ± 1.9 alcoholic drinks, *p* < 0.0001), but over a significantly shorter time (2.1 ± 1.5 vs. 2.8 ± 1.3 h, *p* < 0.0001), resulting in a significantly higher estimated BAC in women (0.036% ± 0.03% vs. 0.028% ± 0.03%, *p* = 0.006). Subjective intoxication during drinking (4.2 ± 2.3 vs. 4.9 ± 2.5, *p* = 0.051) and next morning hangover severity (4.2 ± 2.2 vs. 4.8 ± 2.1, *p* = 0.063) did not significantly differ between men and women. Nonetheless, hangover severity was significantly lower in women than in men, as assessed retrospectively when thinking back on their experience during the walk (2.7 ± 2.1 vs. 3.8 ± 2.4, *p* = 0.001) and after the walk (2.7 ± 2.1 vs. 4.0 ± 2.4, *p* < 0.0001). During the walk, women consumed significantly less water than men (2.3 ± 0.6 vs. 2.9 ± 0.5 L, *p* < 0.0001), but no significant sex differences were found on any walking outcome.

### 3.6. The Association of Water Consumption and Hangover Severity

**Figure 3:**
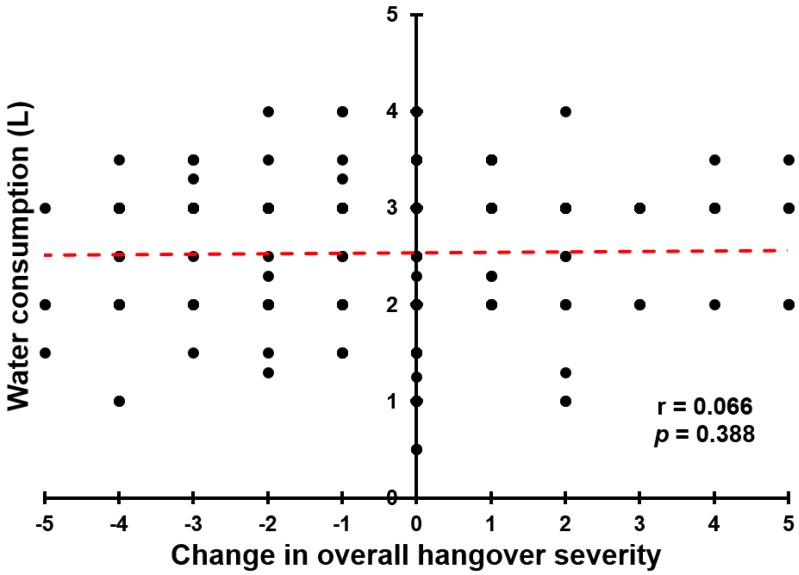
Relationship between water consumption and change in overall hangover severity. A difference score (Δ) was calculated for overall hangover severity, by deducting the morning severity rating from the afternoon severity rating. Red dotted line represents the Spearman’s rho correlation, which was not significant.

In the ‘hangover’ group, the amount of consumed water was significantly correlated with the duration of the walk (*r* = 0.160, *p* = 0.033), the number of breaks (*r* = 0.234, *p* = 0.002), the total duration of the breaks (*r* = 0.173, *p* = 0.022), and the level of reported exhaustion after the walk (*r* = 0.161, *p* = 0.034). Water consumption was not significantly correlated with the amount of effort to complete the walk (*r* = 0.016, *p* = 0.831). No significant correlations were found between the amount of consumed water and overall hangover severity, or other individual hangover symptom scores that were assessed either before, during or after walking the Samaria Gorge. It was further investigated whether the changes in severity scores were associated with the amount of water consumed during the walk. A difference score (Δ, afternoon assessment − morning assessment) was calculated for overall hangover severity and correlated with water intake while walking the Samaria Gorge. The amount of water consumed did not significantly correlate with Δ overall hangover severity (*r* = 0.066, *p* = 0.388) (see Figure 3). The hangover severity difference score between the assessment during walking and the assessment before walking also did not significantly correlate with the amount of water consumed (*r* = 0.103, *p* = 0.174).

### 3.7. The Association of Alcohol Consumption and Smoking after Walking and Hangover Severity

**Figure 4:**
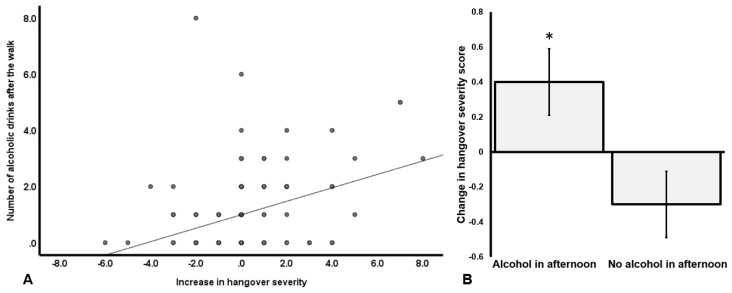
The relationship of alcohol consumption and hangover severity. (**A**) shows the Spearman’s correlation between alcohol consumption and the change in hangover severity (Δ, afternoon assessment − during walking assessment). A positive correlation was found suggesting that higher levels of alcohol consumption during hangover (after the walk) were associated with experiencing more severe hangovers. (**B**) shows the change in hangover severity scores (Δ, afternoon assessment − during walking assessment) for subjects that consumed alcohol after the walk versus subjects that did not consume alcohol after walking the Samaria Gorge. An increase in hangover severity was observed in those who consumed alcohol after the walk, whereas a decrease in hangover severity was observed in subjects who did not consume alcohol after walking the Samaria Gorge. Error bars represent standard errors. The significant difference (*p* = 0.005) between drinkers and those who did not consume alcohol after the walk is indicated by *.

Alcohol consumption after the walk was low across all groups, averaging about one alcoholic drink. However, a consumption range of zero to eight drinks was observed. Similarly, the reported number of cigarettes smoked after the walk varied between zero and 17 cigarettes. For the subgroup that had a hangover in the morning, a difference score (Δ, afternoon assessment − during walking assessment) was calculated for overall hangover severity and correlated with the amount of alcohol consumed and number of cigarettes smoked after walking the Samaria Gorge. A significant correlation was found with the amount of alcohol consumed (*r* = 0.329, *p* < 0.0001), suggesting that alcohol consumption in the afternoon was associated with increased hangover severity (See Figure 4A). The correlation with number of cigarettes smoked after the walk was not significant (*r* = 0.119, p = 0.125). Those who consumed alcohol (*N* = 99) had a significant increase in hangover severity (Δ, afternoon assessment − during walking assessment), mean (SD) + 0.4 (1.9), while subjects that did not consume alcohol (*N* = 75) showed a further decrease in hangover severity after the walk, mean (SD) − 0.3 (1.6) (See Figure 4B). The difference observed change scores of the groups was statistically significant (*p* = 0.005).

When interpreting the data in Figure 4, it should be taken into account that the observed absolute differences are small on a hangover scale ranging from 0 to 10. For the two groups individually, the difference in change scores (hangover severity assessed after walking minus before walking) did not reach statistical significance (*p* = 0.056 and *p* = 0.082 for those who did and did not consume alcohol, respectively). The two groups did not differ in the amount of water consumed during the walk (*p* = 0.199), nor on any of the demographic variables assessed in this study.

## 4. Discussion

The data showed that walking the Samaria Gorge while having a hangover was associated with significantly higher exhaustion scores when compared to participants with no hangover or who consumed no alcohol. However, no significant differences between the groups were found in the duration of the walk, or number and duration of breaks. The amount of water consumed was significantly correlated with the duration of the walk, the number of breaks, the total duration of the breaks, and the level of reported exhaustion after the walk. Further, participants with a hangover consumed significantly more water during the walk. However, neither hangover severity, nor its reduction during the day, were significantly correlated with the amount of water consumed (see Figure 3). The regression analyses revealed that the observed differences in walking outcomes between participants with and without a hangover are related to differences in levels of physical activity in Crete, reported physical fitness, and BMI. The regression analyses revealed that in this sample, sleep outcomes had no relevant impact on walking performance. Furthermore, overall hangover severity did not significantly correlate with any walking outcome and was not a significant predictor in the regression analyses, even though group membership was. The latter suggests that the relative severity of a hangover is not important with regard to endurance performance, but the fact whether or not you experience a hangover per se is a significant determinant. 

As walking the Samaria Gorge is an activity that most people do together with others (e.g., in the current study, several families and groups of friends participated), it is understandable that these individuals all have the same total walking time, number and duration of breaks. Indeed, all three groups completed the walk in approximately the same time, with the same total duration of breaks, providing a useful between groups control. It is therefore understandable that participants with a hangover reported that more effort was needed to perform at this same physical exercise level and reported to be significantly more exhausted than participants who reported no hangover or who had consumed no alcohol. 

Several factors may have influenced our findings. First, this was a naturalistic study, in which the researchers did not intervene with the activities of the participants, nor did they receive any instructions [28]. Participants received no instructions on how to walk the Gorge. The results could have been different if, for example, participants were instructed to walk the Gorge as quickly as possible, or when water intake was restricted to a pre-set volume. Participants were also not aware that they would need to retrospectively rate their hangover symptoms experienced during the walk. Had they known, they might have monitored their subjective experience more closely which might have produced more accurate ratings.

Second, this was a non-student sample of middle-aged adults. As a consequence, this sample consumed much less alcohol in the evening before the walk, as compared to the amounts of alcohol usually seen in hangover studies conducted in student populations [24]. Despite their lower estimated BAC levels, a considerable number of participants reported having a hangover in the morning. Since their weekly alcohol consumption was relatively low, the increase in alcohol consumption seen on holiday in Crete (i.e., about half their usual weekly alcohol intake at home consumed at a single drinking occasion in Crete) may have been sufficient to elicit a hangover, despite the fact that the estimated BAC (0.03%) was well below the levels commonly reported in student samples. This observation supports observations in other studies that lower estimated BAC levels can also elicit a hangover, and that the previously suggested lower limit of 0.11% [29] should be abandoned [30]. 

Third, participants may have anticipated the fact that they had to complete the Samaria Gorge and, as a result, adjusted their drinking and sleep behavior. Perhaps they also practiced for the walk. Although we did not directly assess this with our surveys, the data suggests that overall weekly physical activity levels did not significantly differ between the groups. In contrast, sleep quality was rated as significantly poorer among participants with a hangover. Moreover, a substantial number of participants (8.4%) consumed more alcohol in the evening before the walk than they normally did in a full regular week at home, and on average drinkers consumed about half their usual weekly alcohol intake on this single study occasion in Crete. The latter suggests that the knowledge of having to walk the Samaria Gorge was not a motivation for participants to moderate their alcohol consumption on the evening before the walk. However, the ‘no hangover’ group consumed less alcohol than the ‘hangover’ group. This may suggest that this group did moderate their alcohol consumption in anticipation that they had to walk the Samaria Gorge. 

Interestingly, the ‘no hangover’ group happened to give significantly higher ratings of their immune fitness and physical fitness, as compared to the two other groups. Previous studies have reported a relationship between perceived immune fitness and hangover susceptibility [31], but not with hangover severity [32]. In the current study, hangover severity scores also did not significantly correlate with the immune fitness rating in the ‘hangover’ group. There is anecdotal evidence suggesting that water consumption brings hangover relief. Indeed, the ‘hangover’ group consumed significantly more water than participants from the ‘no hangover’ group, and more than those who consumed ‘no alcohol’. However, the observed reduction in hangover severity during the day was not significantly correlated with the amount of water consumed during the walk. Previous research showed that consumption of water before going to bed did not affect next-day hangover severity [33]. It is important to note that causality cannot be concluded from these correlational analyses. The absence of a significant correlation can either imply that consumption of water is not effective in the relief of hangovers, or that having a hangover does not stimulate individuals to consume more water. We also did not found evidence for the ‘hair of the dog’ effect, i.e., the hypothesis that hangover severity will attenuate by consuming alcohol [34,35]. Instead, the results suggest the opposite, as hangover severity scores increased in those who consumed alcohol in the afternoon and reduced in those who did not consume alcohol. However, these results must be interpreted with caution as, although not very likely, we did not record possible alcohol consumption during the walk. Furthermore, we did not record water and soft drink consumption after the walk. Therefore, future controlled trials are necessary to determine the impact of water and alcohol consumption on the presence and severity of alcohol hangover.

Finally, hangover symptoms were also reported by participants with no hangover, and participants who did not consume any alcohol the evening before walking the Samaria Gorge. The reported symptoms are common complaints, such as sleepiness and thirst, that can be experienced by anyone, including without any specific intervention or event that could explain their occurrence (e.g., alcohol consumption). Some symptoms may be related to sleep loss, whereas other symptoms may be related to the temperature in the Gorge (e.g., the rise in severity scores of thirst while walking), or the physical demands to perform the walk (e.g., increases in scores of weakness during the walk). This points to the importance of using overall hangover severity ratings instead of composite ratings of individual hangover symptoms, as it appears that participants in the ‘no hangover’ group and ‘no alcohol’ group did not attribute their experience of individual symptoms such as sleepiness and thirst to alcohol hangover since their overall hangover severity rating was zero. This finding underlines the importance of including an overall hangover severity score in research design instead of relying on composite symptom scores to calculate an overall hangover score, which is done when using the three most frequently used hangover scales [36,37,38]. 

## 5. Conclusions

In conclusion, this study suggests that a significant hangover may be experienced after relatively low levels of alcohol consumption for this older non-student group. In addition, physical endurance performance was associated with experiencing significantly more exhaustion during the hangover state. Future research should investigate possible hangover effects on other physical activities, such as short-term anaerobic performance (e.g., a 100 m sprint or power lifting) or other forms of long term aerobic exercise (e.g., swimming laps or running long distance). 

## Figures and Tables

**Table 1:** Demographics and physical activity levels of participants with and without a hangover.

Demographics	Overall	No Alcohol	Alcohol, No Hangover	Alcohol, Hangover	*p*-Value
*N*	299	76	47	176	
Age (years)	38.9 (11.0)	39.4 (12.1)	37.5 (11.4)	39.0 (10.3)	0.506
BMI (kg/m^2^)	23.9 (2.4)	23.5 (2.4)	24.4 (2.7)	24.0 (2.4)	0.061
ISQ	8.6 (1.9)	8.8 (1.9)	7.6 (2.5) ‡	8.7 (1.5)	0.006
General health	7.4 (1.1)	7.5 (1.0)	7.9 (1.2)	7.3 (1.0)	0.005
Weekly alcohol consumption at home	4.8 (3.6)	2.0 (1.7)	4.5 (4.0) ‡	5.9 (3.3) *Χ	0.000
**Physical activity at home**					
Vigorous (MET-min/w)	961 (1728)	879 (1937)	1351 (1757) ‡	883 (1617) *	0.035
Moderate (MET-min/w)	1869 (2470)	1928 (3048)	2004 (3868)	1806 (1551)	0.100
Walking (MET-min/w)	3298 (2240)	3511 (1913)	2937 (3725) ‡	3305 (1810) *	0.004
Total (MET-min/w)	5972 (4231)	6124 (4130)	6201 (6008)	5843 (3666)	0.637
Time spent sitting (min)	385 (119)	404 (121)	382 (150)	377 (108)	0.490
**On holiday in Crete**					
Vigorous (MET-min/w)	708 (1432)	823 (1722)	892 (1406)	611 (1299)	0.258
Moderate (MET-min/w)	1588 (1531)	1665 (1766)	1319 (1981)	1627 (1273) *	0.019
Walking (MET-min/w)	3409 (2361)	4099 (2964)	3319 (2953) ‡	3141 (1788) Χ	0.028
Total (MET-min/w)	5588 (3937)	6498 (4569)	5536 (4979)	5219 (4274)	0.155
Time spent sitting (min)	353 (111)	355 (114)	291 (117) ‡	369 (102)	0.000
**Evening before**					
Number of cigarettes smoked	3.7 (5.9)	0.8 (2.8)	1.3 (3.1) ‡	5.6 (6.6) * Χ	0.000
Total sleep time (h)	6.1 (0.8)	6.3 (0.8)	6.2 (1.2)	5.9 (0.6) * Χ	0.000
Number of nightly awakenings	1.1 (1.0)	0.9 (1.0)	1.1 (1.2)	1.3 (0.9) Χ	0.006
Sleep quality	6.1 (1.9)	7.2 (1.9)	7.1 (1.6)	5.4 (1.6) * Χ	0.000

*p*-value from the Kruskal–Wallis test is shown, comparing the outcomes of the three groups. If the group effect was significant (*p* < 0.05), Independent Samples Mann–Whitney U tests were conducted to investigate paired comparisons between the individual groups. Significant differences (*p* < 0.0001) between the ‘hangover’ group and ‘no hangover’ group are indicated by *. Significant differences (*p* < 0.0001) between the ‘no hangover’ group and ‘no alcohol’ group are indicated by ‡. Significant differences (*p* < 0.0001) between the ‘hangover’ group and ‘no alcohol’ group are indicated by Χ. Abbreviations: BMI = body mass index, ISQ = immune status questionnaire, MET = metabolic equivalent of task, min = minutes, /w = per week.

**Table 2:** Drinking characteristics.

	No Alcohol	No Hangover	Hangover	*p*-Value
**Evening before walking**				
Number of alcoholic drinks	0.0 (0.0)	0.9 (1.6)	3.0 (1.8)	0.000 *
Subjective intoxication	---	1.3 (1.9)	4.6 (2.4)	0.000 *
Start time drinking (h.min)	---	19.10 (2.4)	17.40 (1.8)	0.000 *
Stop time drinking (h.min)	---	21.45 (1.9)	20.13 (1.9)	0.000 *
Duration of drinking (h)	---	2.6 (2.4)	2.5 (1.5)	0.157
Estimated BAC (%)	---	0.02 (0.03)	0.03 (0.03)	0.001 *
**Test day**				
Overall hangover severity before walking	---	0.0 (0.0)	4.6 (2.1)	0.000 *
Overall hangover severity during walking	---	1.1 (1.8)	3.4 (2.3)	0.000 *
Overall hangover severity after walking	---	1.1 (2.1)	3.5 (2.4)	0.000 *
**Afternoon after walking**				
Number of alcoholic drinks	0.4 (0.9)	1.0 (1.7)	1.0 (1.3)	0.112
Subjective intoxication	0.6 (1.6)	0.9 (2.1)	1.5 (2.0)	0.010 *
Number of cigarettes smoked	0.4 (1.5)	1.0 (2.3)	3.4 (4.5)	0.000 *

An Independent Samples Mann–Whitney U test was conducted to compare the ‘no hangover’ and ‘hangover’ group. Significant differences between the ‘hangover’ and ‘no hangover’ group (*p* < 0.05) are indicated by *.

**Table 3:** Hangover and symptom severity before, during and after walking the Samaria Gorge.

Group	No Alcohol *N* = 76			No Hangover (in the Morning) *N* = 47			Hangover (in the Morning) *N* = 176		
Symptom	Before	During	After	Before	During	After	Before	During	After
Fatigue	3.6 (2.0)	5.0 (2.4) bd	5.3 (2.3)	2.6 (1.9)	5.9 (2.7) bd	6.1 (2.5) ba	6.1 (1.7) *, Χ	6.3 (1.6) Χ	6.3 (1.7) Χ
Sleepiness	4.0 (2.5)	3.6 (2.5)	4.1 (2.4)	2.5 (2.3)	3.7 (3.1)	4.2 (3.0)	6.5 (2.0) *, Χ	4.8 (2.6) bd	5.3 (2.3) Χ, ba
Thirst	2.1 (2.3)	3.8 (2.8) bd	4.1 (2.9)	2.3 (2.2)	5.1 (2.8) bd	5.1 (2.5) ba	5.6 (2.8) *, Χ	5.1 (2.6)	5.2 (2.6)
Headache	0.7 (1.7)	0.6 (1.4)	1.0 (2.1)	0.4 (0.9)	1.6 (2.6)	0.8 (1.4)	2.2 (3.0) Χ	0.9 (1.7) bd	1.1 (2.0) ba
Nausea	0.3 (0.7)	0.3 (0.9)	0.3 (1.1)	0.1 (0.3)	0.5 (1.2)	0.3 (0.9)	0.8 (1.6)	0.4 (0.9) bd	0.4 (1.3)
Dizziness	0.4 (1.2)	0.4 (1.0)	0.7 (1.5)	1.0 (1.8)	0.6 (1.2)	0.9 (1.7)	1.3 (1.7) *, Χ	0.6 (1.2) bd	0.9 (1.6)
Stomach pain	0.6 (1.4)	0.4 (1.1)	0.7 (1.5)	0.3 (0.8)	0.6 (1.1)	0.8 (1.5)	2.1 (2.2) *, Χ	0.9 (1.5) bd	1.0 (1.4) ba
Heart racing	0.7 (1.6)	1.5 (2.0)	1.2 (2.0)	0.3 (0.7)	2.8 (2.7) bd	2.6 (2.3) ba, ‡	2.3 (2.1) *, Χ	2.1 (2.0)	1.9 (1.7) Χ
Weakness	0.6 (1.2)	2.2 (2.2) bd	2.0 (2.1)	0.5 (0.8)	2.8 (2.1) bd	2.6 (2.5)	2.3 (1.9) Χ	2.9 (1.9)	2.4 (1.9)
Loss of appetite	1.0 (1.9)	NA	0.6 (1.6)	1.4 (1.8)	NA	1.1 (1.6)	1.0 (1.7)	NA	0.5 (1.2)
Anxiety	0.6 (1.1)	NA	0.5 (1.3)	0.7 (1.6)	NA	0.2 (0.6)	1.2 (1.6)	NA	0.8 (1.2)
Depression	0.1 (0.5)	NA	0.2 (0.8)	0.3 (1.0)	NA	0.0 (0.3)	0.3 (1.0)	NA	0.1 (0.5)
Tension, stress	0.6 (1.5)	NA	0.6 (1.7)	0.7 (1.5)	NA	0.5 (1.6)	0.4 (1.0)	NA	0.4 (0.9)
Anger, hostility	0.2 (0.7)	NA	0.3 (0.7)	0.6 (1.5)	NA	0.4 (1.0)	0.3 (0.9)	NA	0.3 (0.7)

Mean (SD) severity scores are shown. A Bonferroni’s correction (*p* < 0.0001) was applied to account for multiple comparisons. Related Samples Wilcoxon Signed Rank tests were conducted to investigate pairwise comparisons between the timepoints of assessment within each group. Significant differences between ‘before walking’ and ‘after walking’ assessments (*p* < 0.0001) are indicated with ‘ba’. Significant differences between ‘before walking’ and ‘during walking’ assessments (*p* < 0.0001) are indicated with ‘bd’. No significant differences between ‘during walking’ and ‘after walking’ assessments (*p* < 0.0001) were observed. Between-group comparisons were made with a Kruskal–Wallis test. If the group effect was significant (*p* < 0.001), Independent Samples Mann–Whitney U tests were conducted to investigate paired comparisons between the individual groups. Significant differences (*p* < 0.0001) between the ‘hangover’ group and ‘no hangover’ group are indicated by *. Significant differences (*p* < 0.0001) between the ‘no hangover’ group and ‘no alcohol’ group are indicated by ‡. Significant differences (*p* < 0.0001) between the ‘hangover’ group and ‘no alcohol’ group are indicated by Χ. Abbreviation: NA = not assessed.

**Table 4:** Performance assessments related to walking the Samaria Gorge.

	No Alcohol	Alcohol, no Hangover	Alcohol, Hangover	*p*-Value
**Assessed before walking**				
Immune fitness	7.5 (1.3)	8.3 (1.5) ‡	7.3 (1.2) *	0.000
Mental fitness	8.6 (1.3)	8.6 (1.2)	8.5 (1.2)	0.628
Physical fitness	6.9 (1.5)	7.4 (1.8)	6.8 (1.4)	0.057
Expected effort to walk the Gorge	8.0 (1.9)	6.6 (1.9) ‡	8.4 (1.4)	0.000
**Assessed after walking**				
Immune fitness	6.9 (1.2)	6.8 (1.7)	6.3 (1.4) Χ	0.003
Mental fitness	7.9 (1.4)	7.8 (1.7)	7.7 (1.5)	0.636
Physical fitness	6.8 (1.3)	6.4 (1.8)	6.1 (1.4) Χ	0.004
Effort to walk the Gorge	8.1 (1.4)	7.9 (1.7)	8.3 (1.5)	0.135
Exhaustion	8.3 (1.4)	7.8 (2.2)	8.8 (1.4) * Χ	0.002
Walking time (hours)	6.1 (0.7)	6.1 (0.9)	6.0 (0.6)	0.734
Number of breaks	4.3 (1.7)	4.4 (1.9)	4.0 (1.1)	0.666
Total duration of breaks (minutes)	24.3 (12.5)	26.7 (19.0)	25.6 (8.7)	0.227
Water consumed during the walk (liters)	2.3 (0.9)	2.4 (0.8)	2.7 (0.7) * Χ	0.001

*p*-value from the Kruskal–Wallis test is shown, comparing the outcomes of the three groups. If the group effect was significant (*p* < 0.05), Independent Samples Mann–Whitney U tests were conducted to investigate paired comparisons between the individual groups. Significant differences (*p* < 0.0001) between the ‘hangover’ group and ‘no hangover’ group are indicated by *. Significant differences (*p* < 0.0001) between the ‘no hangover’ group and ‘no alcohol’ group are indicated by ‡. Significant differences (*p* < 0.0001) between the ‘hangover’ group and ‘no alcohol’ group are indicated by Χ.

**Table 5:** Correlates of walking performance.

	Predictive Validity of the Model	Contribution of Individual Variables
Exhaustion	11.0%	Physical fitness rate before walking (6.3%) Group (2.6%) Physical activity (total METs) in Crete (2.1%)
Effort	29.6%	Physical activity (total METs) in Crete (14.5%) Physical fitness rate before walking (6.7%) Number of cigarettes smoked (3.4%) Group (1.9%) Number of alcoholic drinks consumed (1.6%) BMI (1.5%)
Duration of the walk	29.2%	Physical fitness rate before walking (11.0%) Physical activity (total METs) in Crete (5.1%) BMI (3.7%) Past year’s immune status (ISQ) (3.7%) Usual weekly alcohol consumption (3.0%) Time spent sitting on a week day in Crete (2.7%)
Number of breaks	19.9%	Time spent sitting on a week day at home (7.9%) BMI (3.7%) Physical activity (total METs) in Crete (3.5%) Sex (2.9%) Group (1.9%)
Total duration of breaks	17.5%	Usual weekly alcohol consumption (6.5%) BMI (6.2%) Duration of drinking evening before (4.8%)
Amount of water consumed	21.1%	Sex (15.1%) Physical activity (total METs) in Crete (7.2%) Past year’s immune status (ISQ) (5.2%) BMI (3.2%) Physical fitness rate before walking (1.4%)

The regression models show that physical activity (total METs) in Crete, the physical fitness rate before walking, and BMI are important factors that predict walking outcomes. Albeit modest, ‘group’ membership (‘no alcohol’, ‘no hangover’, ‘hangover’) significantly predicted exhaustion after the walk, effort to perform the walk, and the number of breaks. Sex had the largest impact on water consumption.
